# Transcriptomic analysis reveals responses to Cycloastragenol in *Arabidopsis thaliana*

**DOI:** 10.1371/journal.pone.0242986

**Published:** 2020-12-10

**Authors:** Wissem Mhiri, Merve Ceylan, Neslihan Turgut-Kara, Barbaros Nalbantoğlu, Özgür Çakır

**Affiliations:** 1 Chemistry Department, Faculty of Art & Science, Yıldız Technical University, Istanbul, Turkey; 2 Program of Molecular Biology and Genetics, Istanbul University, Institute of Science, Istanbul, Turkey; 3 Department of Molecular Biology and Genetics, Faculty of Science, Istanbul University, Istanbul, Turkey; East Carolina University, UNITED STATES

## Abstract

Cycloastragenol (CAG), a molecule isolated from ‘*Astragalus membranaceus’*, stimulates the telomerase activity and cell proliferation significantly. It has been proven that CAG has the ability to prevent some diseases in humans. In this study, we aimed to figure out the CAG effects on the different signaling mechanisms in plants and to broadly analyze the genome-wide transcriptional responses in order to demonstrate CAG as a new key molecule that can potentially help plants to overcome different environmental stresses. RNA-seq strategy was employed to assess the transcriptional profiles in *A*. *thaliana* calli. Our work primarily focused on an overall study on the transcriptomic responses of *A*. *thaliana* to CAG. A total of 22593 unigenes have been detected, among which 1045 unigenes associated with 213 GO terms were differentially expressed and were assigned to 118 KEGG pathways. The up-regulated genes are principally involved in cellular and metabolic processes in addition to the response to a stimulus. The data analysis revealed genes associated with defense signaling pathways such as cytochrome P450s transporter, antioxidant system genes, and stress-responsive protein families were significantly upregulated. The obtained results can potentially help in better understanding biotic and/or abiotic tolerance mechanisms in response to CAG.

## Introduction

Extracted from *Astragalus* roots, a plant common to be used in Chinese traditional medicine, Cycloastragenol (CAG, molecular weight: 490,72 g/mol) is known to play a significant role in telomerase activation in Eukaryotic cells which is tightly linked to genome stability and involved in the prevention of some human diseases. Cycloastrogenol enhances the function of the immune system and has several pharmacological actions such as anti-aging, anti-inflammation, anti-fibrosis, anti-bacterial, and liver protection. Cycloastrogenol stimulates urinary and circulatory systems in humans and helps to kill cancer cells by promoting the immunotherapy of some types of cancers, and it helps to heal burns and prevent various heart diseases [1, 2]; it ameliorates cardiac dysfunction and remodels through promoting autophagy in myocardial cells [3] and reduces depression-like behavior [4]. A complete apperception of these facts would provide practical fulfillment for plants since they, like humans, undergo different kinds of stress, biotic and abiotic, which lead to different biochemical, physiological molecular and gene expression changes. In the literature, there is no study evaluating the effect of CAG on plants. However, CAG would be a key molecule to develop our understanding of genes that could help plants to overcome some stress limits. New approaches are required to help to figure out CAG-affected pathways, highlighting the involved genes and their expression profiling, which will promote the understanding of the concerned mechanisms. The availability of *Arabidopsis thaliana* genome sequence and the development of Next Generation Sequencing technologies allow identifying and analyzing essential genes at the transcriptional level. Genomic analyses and the study of gene functions in the model and non-model organisms come to be much easier nowadays thanks to the advances in sequencing technologies. NGS is one of the most useful strategies investigating plant transcriptome in certain conditions; however, among NGS technologies, RNA-seq is the most powerful tool to compare transcriptome profiling. It is a rapid, highly reproducible, and a low-priced whole-transcriptome analysis procedure enabling us to analyze transcriptomes and to understand the expression profile of the genome [5–7]. RNA-seq has been used in plants in order to study diverse environmental-responses; the transcriptional changes in roots [8] and leaves [9] of *A*. *thaliana*, have been studied under Fe deficiency. Two stresses; heat and drought from one side and cold and high light intensity from another side were combined to study the responses of the transcriptome in the same model plant [10]. Moreover, a transcriptomic analysis was made in *A*. *thaliana* colonized by a *Rhizobacterium* infection [11]. In the present study, the aim is to resolve the effect of CAG on the different pathways in *A*. *thaliana* and to study the expression of the involved gene(s), which might provide useful knowledge to establish breeding programs for pharmaceutical and/or agricultural purposes. We analyzed CAG-treated calli from *Arabidopsis* roots by high-throughput RNA-Seq library. Transcriptional network and the main metabolic activities involved in CAG treatment consequences on plant growth and their behavior towards environmental stresses were determined based on a transcriptomic data analysis using bioinformatics tools. Transcriptome results were validated using a quantitative real-time PCR (q-RTPCR) analysis. Our study provides a broad-spectrum study of the *A*. *thaliana* transcriptomic response to Cycloastragenol and a clarification of the differentially expressed genes in *Arabidopsis* calli.

## Material and methods

### Plant material, seed sterilization, media preparation, and callus formation

Wild type, Col-0, *Arabidopsis thaliana* seeds were supplied by Dr. Ralf Stracke from the University of Bielefeld. Seeds were surface sterilized with 70% Ethanol and sown on Petri dishes filled with MS basal medium, containing 3% (w/v) sucrose, solidified with 0.9% (w/v) agar and adjusted to pH 5.7. Petri dishes were incubated in the plant growth chamber under fluorescent light (16h light/8h dark light conditions) at 25°C for 14 days until germination. The seedling was transferred then on test tubes filled with MS medium for the two following weeks, and the 30 days old plants were used for callus formation. Root explants (≈ 1 cm) of 30 days old plants were excised from the plants and surface sterilized. They were aseptically cut and placed in contact with the wounded area into fresh MS medium containing 0.5 mL of 2,4-dichlorophenoxyacetic acid (2,4-D). Callus cultures were continuously sub-cultured for 9 months.

### Cycloastragenol treatment

Callus tissues obtained from these explants were subcultured 3-week intervals for 9 months by transferring to fresh MS medium containing 1 mg/L 2,4-D. calli were divided into two different groups based on the supplemented CAG concentration; Control group, 1 μM CAG. For each concentration, three biological repetitions have been set. Calli were developed in a plant growth cabin at 25°C ±1°C and under 16h light/8h dark of light conditions for 21 days. Samples were collected for morphological analysis and then stored at -80°C until being used for RNA extraction.

### RNA isolation

Total RNA was isolated from the frozen calli of *A*. *thaliana* using TRIzol reagent (Invitrogen, USA). RNAs integrity was assessed by Nanodrop 2000 Spectrophotometer (Nanodrop Technologies, USA). All RNAs extracted from each group were pooled in an equimolar amount to have two sets of total RNAs, one Control and 1μM CAG, ready for cDNA library construction.

### Library construction and sequencing

Fragmentation of mRNA, cDNA libraries construction and sequencing were carried out by the Beijing Genomics Institute (BGI, Shenzhen, China). Corresponding to three biological replicates from each group, libraries were constructed following the Illumina High-Throughput sequencing library protocol. After checking its integrity, 40μg of total RNA is used to purify the poly-A containing mRNA molecules using poly-T oligo attached magnetic beads and shearing it into small pieces using divalent cations under high temperature. Fragments of mRNA were used as a template to synthesize cDNA. A single ‘A’ base and subsequent ligation of the adaptor were added, and a PCR was performed. PCR products were quantified by Qubit, and samples were then pooled to make a single strand DNA circle (ssDNA circle), which gave the final library. DNA nanoballs (DNBs) were generated with the ssDNA circle by rolling circle replication (RCR) to enlarge the fluorescent signals at the sequencing process. DNBs were read through on the BGISEQ-500 platform for the following data analysis study.

### Transcriptome studies and functional annotation

Reads with adaptors, the low-quality raw reads (the percentage of the base which quality is lesser than 15 is greater than 20% in a read) and reads with unknown bases (N bases more than 5%) were filtered using the software SOAPnuke (v1.5.2, https://github.com/BGI-flexlab/SOAPnuke) [12] in order to obtain the clean reads which were stored in FASTQ format. Clean reads were mapped using HISAT (Hierarchical Indexing for Spliced Alignment of Transcripts, v2.0.4, http://www.ccb.jhu.edu/software/hisat) [13] and transcriptome assembly was set using StringTie software (v1.0.4, http://ccb.jhu.edu/software/stringtie) [14] and then compared to the reference annotation (the known genes of *A*. *thaliana* genome) using Cufflinks tools Cuffcompare (v2.2.1, http://cole-trapnell-lab.github.io/cufflinks) [15].

### Quantification of gene expression

The clean data were mapped to the unigene library using **Bowtie2** (v2.2.5, http://bowtie-bio.sourceforge.net/Bowtie2/index.shtml) [16], then the expression level for each gene from our data is measured using **RSEM** (v1.2.12, http://deweylab.biostat.wisc.edu/RSEM) [17] and FPKM (fragments per kilobase per million fragments mapped) for each unigene was calculated to determine the unigene expression profiles. Genes with differential expression (DEG) between the two samples were identified via PossionDis [18], and the results were corrected for multiple testing with the Benjamini–Hochberg false discovery rate (FDR) of adjusted p-value ≤ 0.05, using the parameters **FDR** > = 2.00 and **FDR** < = 0.001 followed by their hierarchical clustering and their Gene Ontology analysis using **phyper, a function of R**. The selected DEGs were compared to the Kyoto Encyclopedia of Genes and Genomes (KEGG) pathway database and Gene Ontology for respectively pathway mapping and GO-term enrichment. To obtain a more general overview of changes in gene expression in the involved pathways, the DEGs were also analyzed. Actually, to find the ORF of each DEG, **getorf** (EMBOSS: 6.5.7.0, http://www.bioinformatics.nl/cgi-bin/emboss/help/getorf) which were aligned to the TF domain by using **PlntfDB** (Version: v23.0, http://plntfdb.bio.uni-potsdam.de/v3.0/) [19].

### Quantitative real-time PCR analysis

To confirm the RNA-Seq results, seven genes were randomly selected to test gene expression levels using real-time qPCR. Primer sets were designed with the Primer3 online software (S1 Table). 20μL reaction systems were analyzed in triplicate; each reaction mixture contained 10 μL of 1 × SYBR Green Supermix (Hibrigen, Turkey), 2 μL of first-strand cDNA obtained from the same RNA samples mentioned above using the High Capacity cDNA Reverse transcription kit from (Applied Biosystem^TM^, USA) 1 μL of the forward primer and 1 μL of reverse primer (10 μmol/L). The qRT-PCR program was as follows: denaturation at 95°C for 5 min, followed by 45 amplification cycles of 95°C for 15 s, and Depending on the primer a determined temperature by gradient PCR, is run for 60 s, then 72°C for 30 s. A melting curve was generated to verify the specificity of the amplification (from 65 to 95°C with an increment of 0.5°C per cycle, with each cycle held for 5 s). Expression of *A*. *thaliana* actin (AT3G18780) was used to normalize the expression of the genes in each corresponding qRT-PCR sample using actin-specific primers (S1 Table). Quantitative variation between different samples was calculated using the delta delta cycle threshold method (ΔΔct).

## Results and discussion

In the present work, *A*. *thaliana* Col-0 calli were grown on medium supplied without and with Cycloastrogenol. Our preliminary results showed that CAG induce an increase of growth index of our calli after measuring it before and after treatment. Even though a non-significant increase has been observed, these results incite us to suggest that it is of interest to investigate the CAG-induced physiological responses. To broadly analyze the molecular and the physiological mechanisms and the genome-wide transcriptional responses caused by Cycloastragenol, we sequenced two cDNA libraries developed from *A*. *thaliana* (wild type Col-0) treated calli with 1μM and without CAG.

The sequenced dataset generated in the present study has been deposited at the Gene Expression Omnibus (GEO) and the Sequence Read Archive (SRA) databases of National Center for Biotechnology Information (NCBI) under the accession numbers, respectively, GSE158409 and SRP285089.

### High throughput sequencing and quality filtering

**Fig 1:**
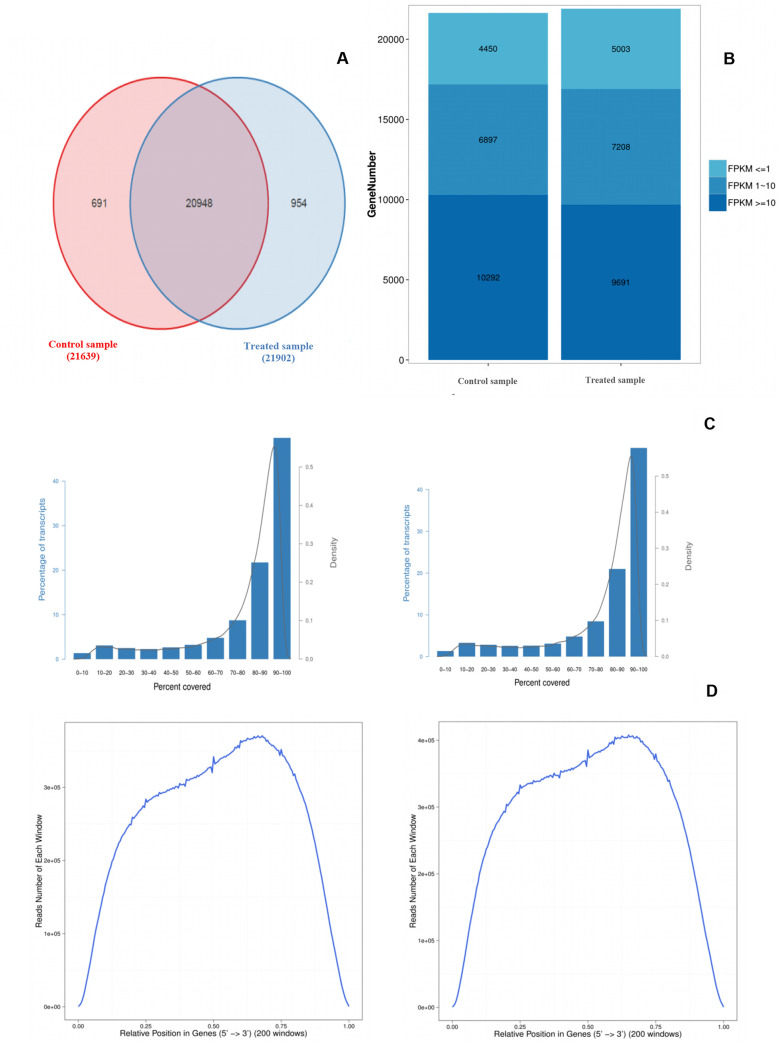
Venn diagram, gene expression distribution, reads coverage and distribution analysis of transcripts. **(A)** Venn diagram displaying expressed genes between control and treated sample: Sample_1A: Control, Sample_1B: CAG treated. **(B)** Gene expression distribution showing the gene amount under different FPKM value: X axis represents the sample name. Y axis represents the gene amount. The dark color means the high expression level which FPKM value ≥ 10, while the light color means the low expression level which FPKM value ≤ 1. **(C)** Read coverage of each detected transcripts. X axis represents the reads coverage. Y axis on left side represents the percentage of transcripts. Y axis on right side represents the density of transcripts. Control sample on the left, treated sample on the right. **(D)** Read distribution of each detected transcript. X axis represents the position along transcripts. Y axis represents the number of reads. Control sample on the left, treated sample on the right.

Two libraries were constructed using total RNA isolated from CAG-treated and non-treated calli. High throughput sequencing of transcriptome libraries using BGISEQ-500 platform generated an aggregate of 133.586.630 raw reads, presenting 63.509.590 and 70.077.040 respectively for control and CAG-treated samples. To determine the certainty of the data analysis, the original sequences were filtered to establish the quality of each read. Reads which had adapter, low base quality (≤ Q20%), sequences with N content less than 5%, and rRNA sequences were removed to retain 56.215.604 and 62.620.022, representing about 48% and 52% of clean raw reads for, respectively, control and CAG-treated libraries. The Q20 value of the reads was over 96% for both samples (Table 1). As a result, around 11.88 GB clean bases were generated from our two libraries, about 5,94GB per sample. The average genome mapping rate is 96.72%, and the average gene mapping rate is 91.94%. Mapped clean reads and calculated expression gene by **RSEM** software showed a total of 43.541 gene numbers about 21.639 and 21.902 genes (Fig 1A), corresponding to 33.972 and 34.193 transcripts, respectively, for control and CAG-treated sample as it shown in Table 1. The calculation of the amount of gene under three different FPKM (Fragments Per Kilobase Million) values (FPKM ≤ 1, FPKM 1~10, FPKM ≥ 10) is provided in Fig 1B. Between the two samples, more than 10,000 genes with an FPKM value ≥ 10 were found in treated and non-treated samples. Reads coverage and reads distribution of each transcript is shown in Fig 1C and 1D presenting their percentages and their density. The distribution of the gene expression level of each sample showed a disruption.

**Table 1:** Filtering of raw reads obtained through high throughput sequencing of RNA-Seq libraries.

Sample	Total raw reads	Total Clean Reads	Total Clean Bases	Clean reads Q20	N reads ratio	Clean Reads Ratio	Total Gene Number	Total Transcript Number
Control	63.509.590	56.215.604	5.621.560.400	96.4%	0,00%	88.51	21.639	33.972
1μM CAG	70.077.040	62.620.022	6.262.002.200	96.55%	0,00%	89.36	21.902	34.193

Sample: sample name, total raw reads (Mb): The reads amount before filtering, unit: Mb, total clean reads (Mb): The reads amount after filtering, unit: Mb, total clean bases (Gb):, The total base amount after filtering, unit: Gb, clean reads Q20 (%): The Q20 value for the clean reads, N reads ratio: The total amount of reads which contain more than 5% unknown N base, clean reads ratio (%): The ratio of the amount of clean reads, total gene number: The amount of all genes, total transcript Nnmber: The amount of all transcripts

### RNA-Seq global data analysis and evaluation of differential gene expression

**Fig 2:**
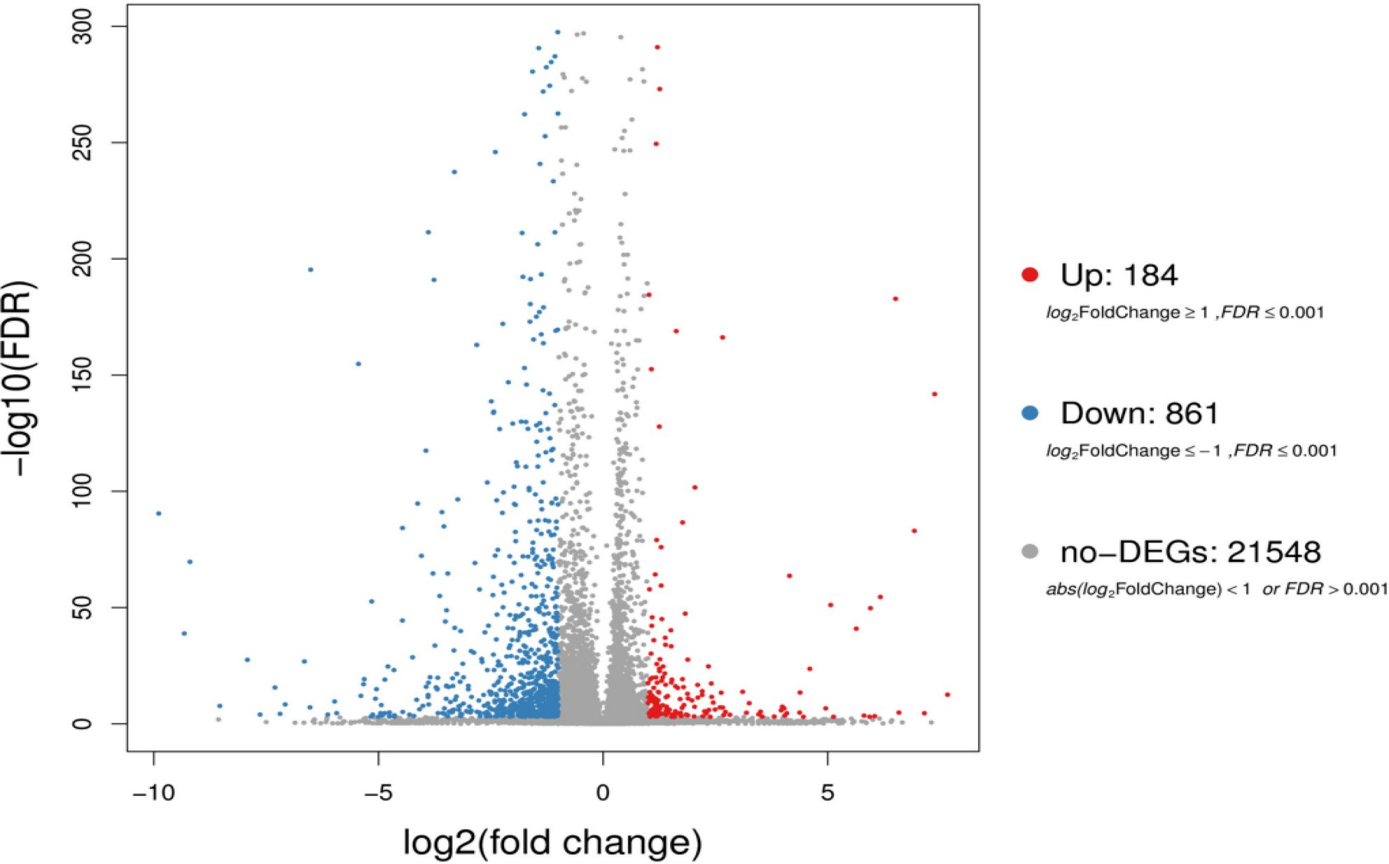
Volcano plots of differentially expressed genes (DEGs). Summary of DEGs is shown in volcano plot. X axis represents log2 transformed fold change. Y axis represents -log10 transformed significance. Red points represent up-regulated DEGs. Blue points represent down-regulated DEGs. Gray points represent non-DEGs.

To figure out the effect of Cycloastrogenol on gene expression of *A*. *thaliana*, we determined the Differentially Expressed Genes (DEGs) between control and CAG-treated callus. Based on the gene expression level, DEGs are identified using PossionDis algorithms. To present an overview of these genes, a volcano plot (Fig 2) has been used to demonstrate their overall distribution. In the volcano plot, red and blue dots represent significant differentially expressed genes, while grey pots represent non-significantly differentially expressed ones. Actually, the total number of genes found to be differently expressed upon to CAG application in *Arabidopsis* is 22593 which have been filtered to be 1045 DEGs; CAG-treatment significantly up-regulated the expression of 184 genes (FDR ≤ 0.001; log2FoldChange ≥ 1), while it significantly down-regulated the expression of 861 genes (FDR ≤ 0.001; log2FoldChange ≤ -1). The size distribution of the filtered DEGs is as following; DEGs are divided into 5 groups; 30 unigenes shorter than 500 pb, 191 unigenes between 500 et 1000 pb, 607 unigenes with a size between 1000 and 2000 pb and 217of unigenes longer than 2000 pb.

### Gene Ontology (GO) analysis of RNA-seq data

**Fig 3:**
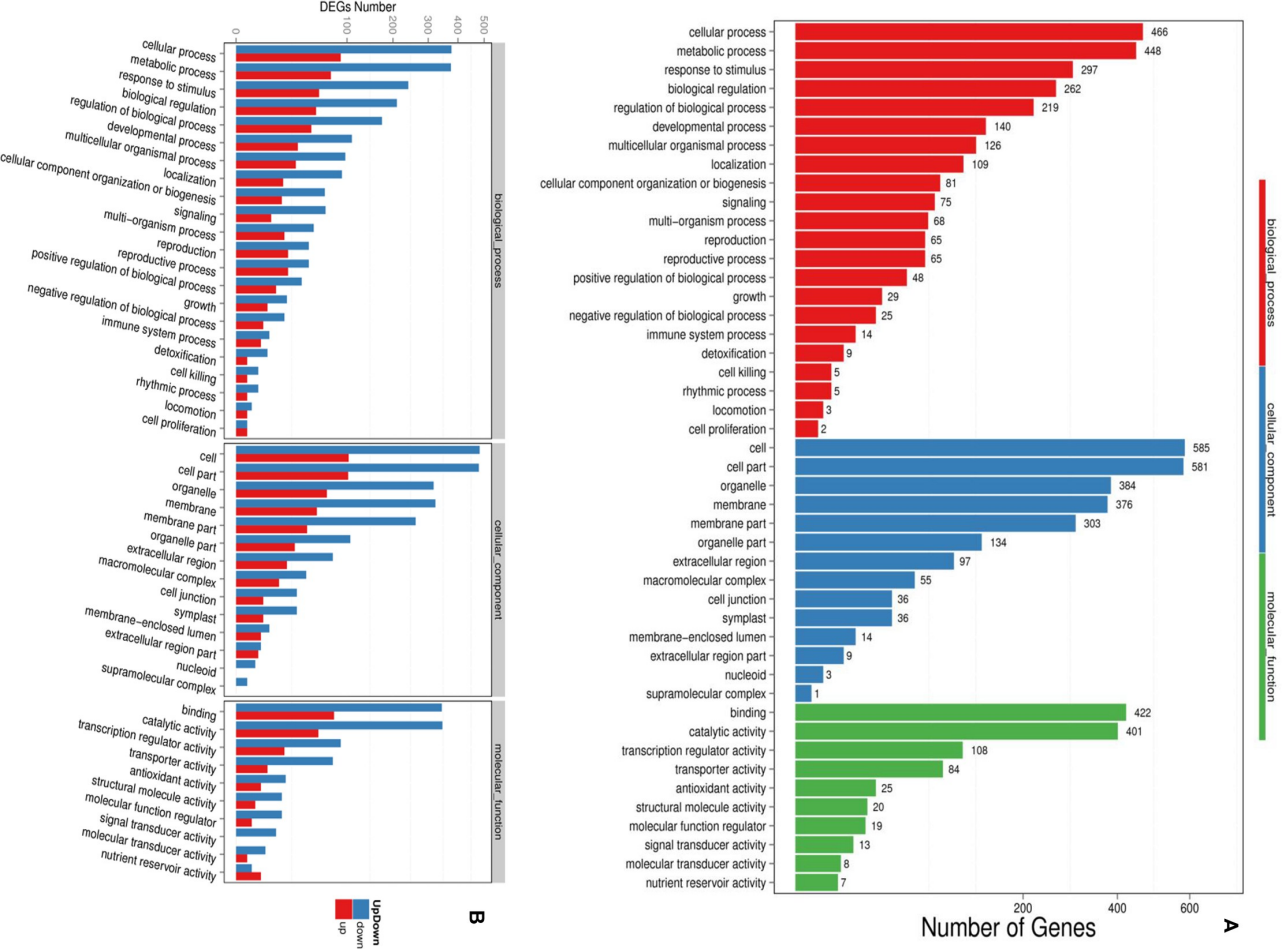
Gene ontology analysis of DEG. The GO classification results are shown. **(A)** GO has three ontologies: molecular biological function, cellular component and biological process. X axis represents number of DEG. Y axis represents GO term. **(B)** The GO classification of up-regulated and down-regulated genes. X axis represents GO term. Y axis represents the amount of up/down-regulated genes.

The list of differentially expressed genes (DEGs) was analyzed for enrichment in Gene Ontology (GO) classification and functional enrichment using **AmigoGO2**. GO Analysis has been done in order to inspect the mechanisms that these genes might belong to and divided all genes into three major groups; molecular function, cellular component, and biological process. Based to this analysis, DE genes have been profoundly enriched for many GO process and function categories. Actually, among the 1045 DEGs, gene number associated to one or more GO terms is different based on the functional category. All genes must be associated with at least one GO term. However, some genes were not annotated to any GO term. A total of 1754 GO terms were associated with all genes and arranged in 46 annotated functional subcategories (Fig 3A). The majority of the GO annotations belongs to the Biological processes; 1139 GO terms (672 genes, 64% of all DEGs) followed by molecular functions which include 427 GO terms (707 genes, 67% all DEGs) and then the cellular components including 188 GO terms the (757 genes, 72% of all DEGs). Among these categories, twelve subcategories are found to be the most abundant; cell (GO: 0005623), cell part (GO: 0032990), metabolic process (GO: GO:0008152), cellular process (GO: 0009987), binding (GO: 0005488), catalytic activity (GO: 0003824), membrane (GO: GO:0016020), organelle (GO: 0043226), membrane part (GO: 0048501), response to the stimulus (G0: GO:0050896), biological regulation (GO: 0065007) and regulation of biological process (GO: 0050789). The GO classification of up-regulated and down-regulated genes show that both are distributed on the three ontologies. However, most of them belong to cell and cell part GO terms. The most enriched GOs of DE genes were shown in Fig 3B. In biological process category, cellular process, metabolic process, and response to stimulus ontologies were the top three gene ontology terms with the number of unigenes 466, 448, and 297, respectively. In the same category, the rhythmic process, locomotion, and cell proliferation represent the last three GO terms with, respectively, 5, 3, and 2 unigenes. In the cellular component category, cell, cell part, and organelle terms were the top three terms with 585,581 and 384 unigenes. The last three categories were extracellular region part, nucleoid, and supramolecular complex with a number of unigenes of 9, 3, and 1, respectively. For the molecular function category, binding with 422, catalytic activity with 401 and transcription regulator activity with 108 unigenes are the top three GO terms. Signal transducer activity, molecular transducer activity, and nutrient reservoir activity represent the least three GO terms with 13, 8, and 7 unigenes, respectively (Fig 3A). The obtained data brings useful information about gene expressions and functions in plant growth, metabolism and stress resistance under CAG treatment. It shows how metabolism of *A*. *thaliana* is affected by Cycloastragenol.

### KEGG analysis

**Fig 4:**
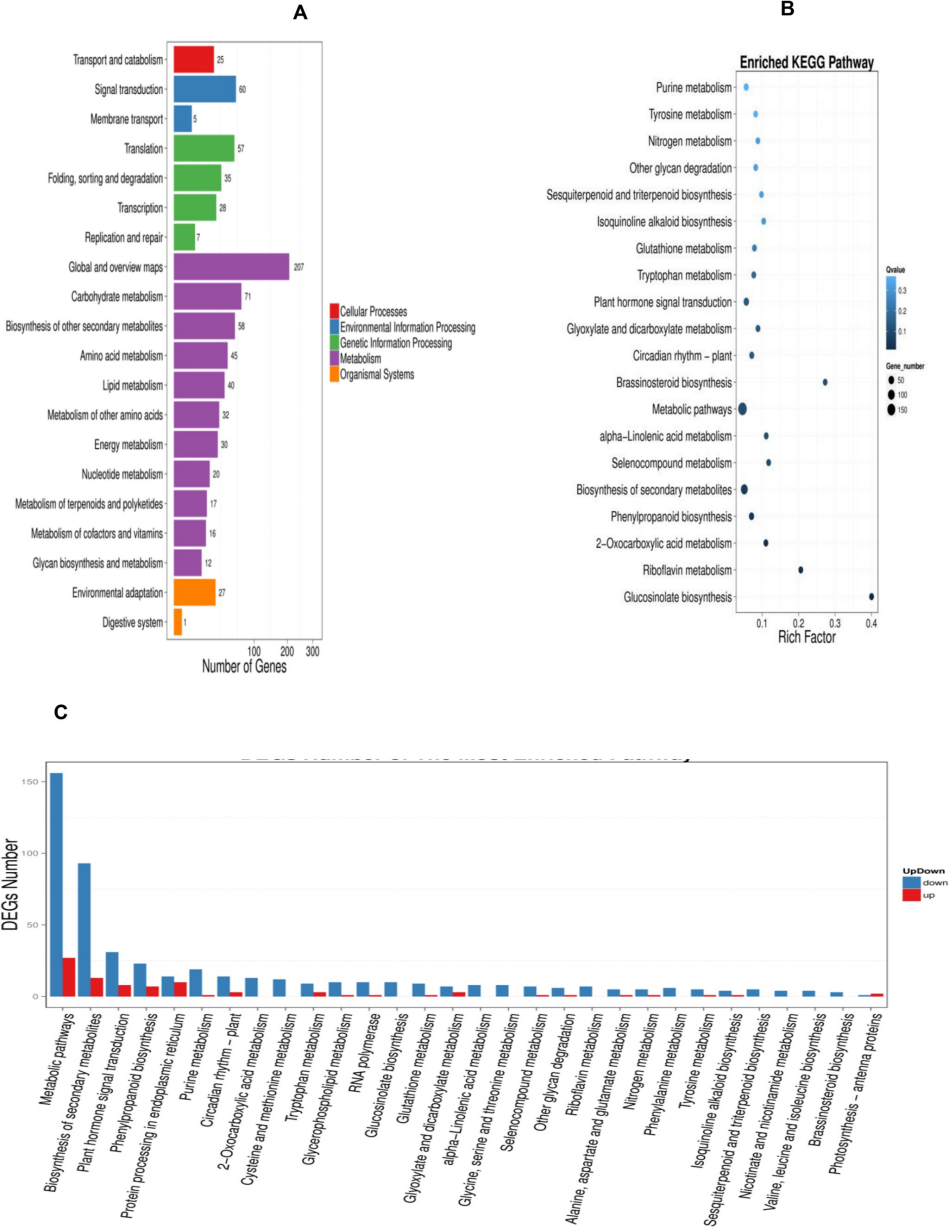
KEGG pathway classification and functional enrichment. **(A)** The pathway classification of DEGs results. X axis represents number of DEG. Y axis represents functional classification of KEGG. **(B)** The pathway functional enrichment results. X axis represents enrichment factor. Y axis represents pathway name. The color indicates the q-value (high: white, low: blue), the lower q-value indicates the more significant enrichment. Point size indicates DEG number (The bigger dots refer to larger amount). **(C)** The pathway functional enrichment result for up/down regulation genes. X axis represents the terms of Pathway. Y axis represents the number of up/down regulation genes.

**Fig 5:**
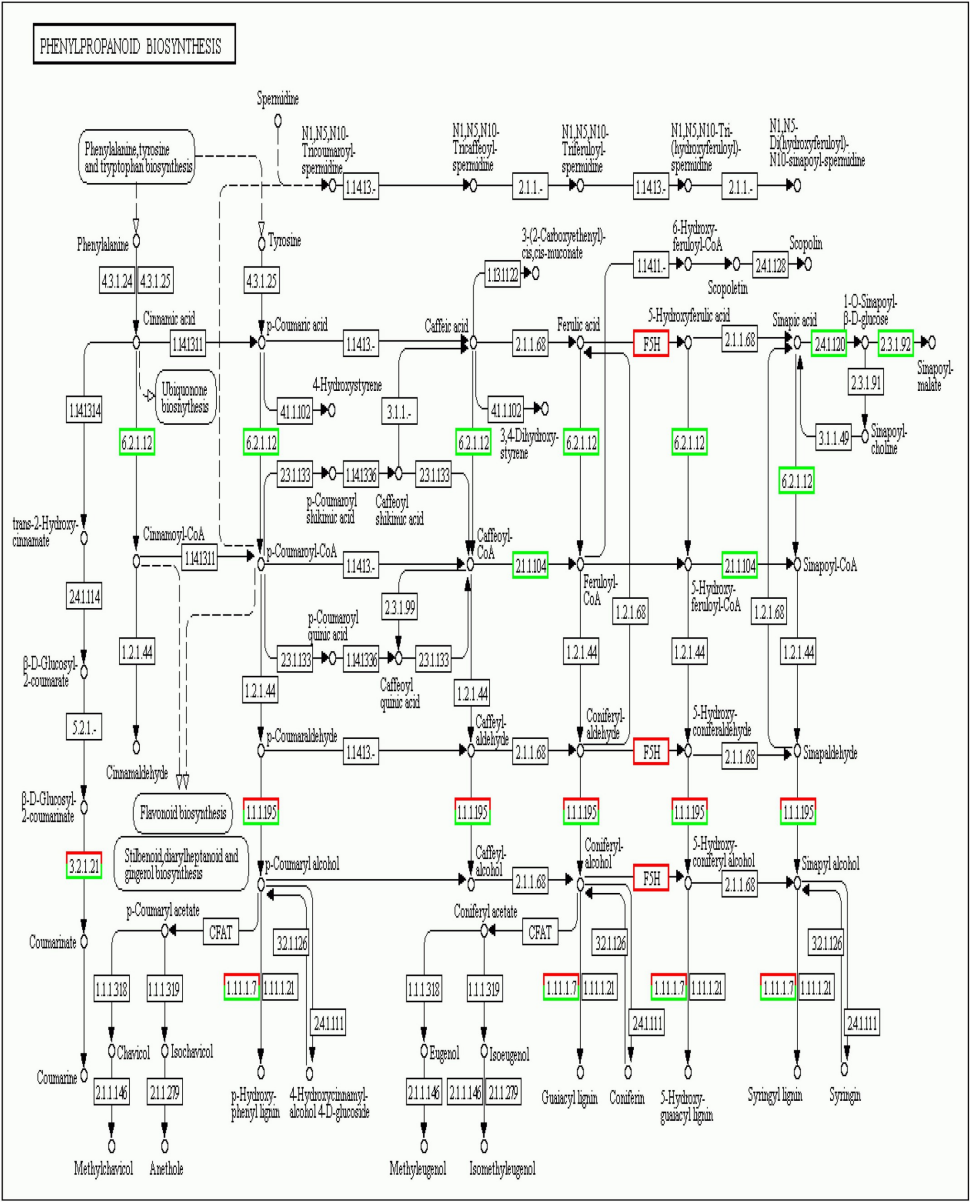
Phenylpropanoid biosynthesis pathway in CAG-treated *A*. *thaliana* calli. Red boxes and green boxes represent up-regulated and down-regulated genes, respectively.

**Fig 6:**
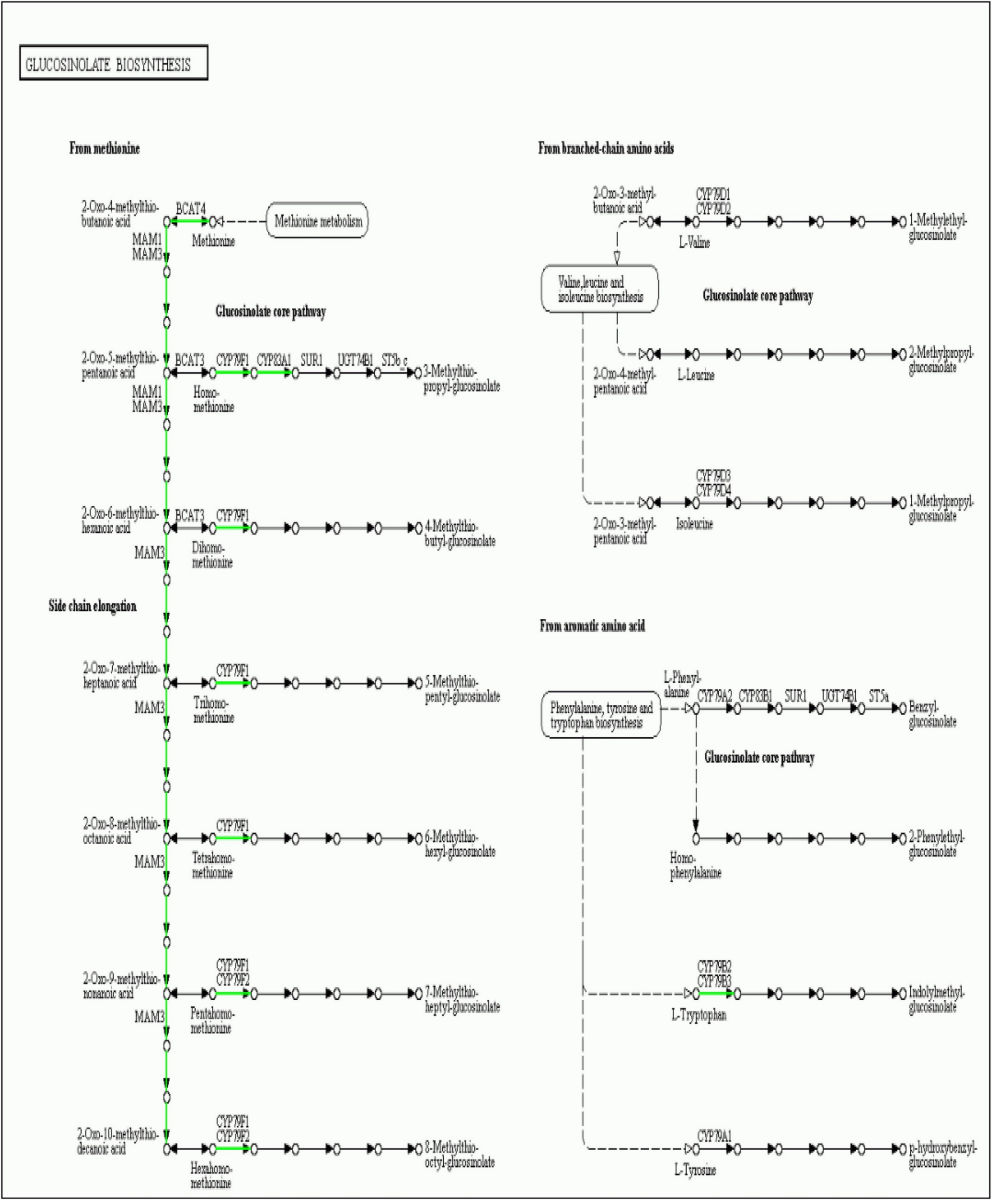
Glucosinolate biosynthesis pathway in CAG-treated *A*. *thaliana* calli. Green lines represent down-regulated genes.

For KEGG (Kyoto Encyclopedia of Genes and Genomes) analysis, 20.101 unigenes were annotated with the pathway analysis, and it was determined that 784 from them were assigned to KEGG pathways. The KEGG pathways have been given based on the ratio of genes enriched to the concerned pathways, on *P-values* and *Q-values* (corrected P-value) both indicate the degree of enrichment and the smaller they are, the more significantly DEGs enriched the pathway. The most significantly enriched pathways in our study are ‘Glucosinolate metabolism’, ‘Riboflavin metabolism’, ‘2-Oxocarboxylic acid metabolism’ and ‘Phenylpropanoid biosynthesis’ pathways with a *P-value* of 0.00000001477425, 0.0002860938, 0.0007007765 and 0.00130124, respectively (Table 2). To perform the pathway and functional classification of *Arabidopsis* DEGs induced by CAG treatment, DEGs were analyzed to the KEGG database and were assigned to 118 pathways. The most enriched pathways with the corresponding number of the involved DEGs were listed in Fig 4A. The 118 pathways were categorized in 5 biological function classifications; Cellular processes, Environmental information processing, Genetic information processing, Metabolism and Organism systems. Actually, most of the pathways are related to metabolism, with 548 differentially expressed unigenes. Genetic Information Processing and Environmental Information Processing are the second and the third categories with, respectively, 127 and 65 differentially expressed genes. The Rich Factor, which is the quotient of foreground value (the number of DEGs) and background value (the total Gene amount), show that metabolic pathways, biosynthesis of secondary metabolites, and Plant hormone signal transduction are the most enriched functional pathways (Fig 4B). The enrichment analysis of DEGs pathway significance gives the detailed information, performing the maps of each pathway with the involved up and down-regulated genes (Fig 4A and 4B), pathway functional enrichment for those genes results are shown in Fig 4C. Based on the number of involved DEGs (Table 2), the most abundant KEGG pathways in our study are ‘Metabolic pathways’ (23.34%, ko01100, no map in kegg), ‘Biosynthesis of secondary metabolites’ (13.52%, ko01110, No map in kegg) and ‘Plant hormone signal transduction’ (4.97%, ko04075, S1 Fig). In the plant hormone signal transduction, genes coding SAUR-like auxin-responsive protein family, EXS (ERD1/XPR1/SYG1) family protein, squamosa promoter binding protein-like 4, basic helix-loop-helix (bHLH) DNA-binding superfamily protein and response to ABA and SALT1 were induced by CAG. Some other pathways, such as ‘Phenylpropanoid biosynthesis’ (3.83%, ko00940, Fig 5), ‘MAPK signaling pathway-plant’ (3.06%, ko04016, S2 Fig),’Starch and sucrose metabolism’ (2.81%, ko00500, S3 Fig) and ‘Protein processing in endoplasmic reticulum’ (3.06%, ko04141, S4 Fig) had also a significant portion of DEGs with pathway annotation. Genes related to Phenylpropanoid biosynthesis (3.83%, ko00940, Fig 6) involved in the synthesis of phenolic acids and known to be one of the most significant secondary metabolisms in plants, and starch and sucrose metabolism, which plays a role in carbohydrate metabolism, have been reported to be associated with cell wall formation [20, 21]. Glucosinolate metabolism (1.28%, ko00966) has a significant part of DEGs with pathway annotation, it is related to metabolism processing, and one of the most important secondary metabolism pathways in plants since a lot of studies have been studying roles of glucosinolates and glucosinolate-derived metabolites in plant metabolism, growth, and their defense mechanisms.

**Table 2:** Enriched pathway functional results.

Pathway	DEGs with pathway annotation (784)	All genes with pathway annotation (20101)	P-value	Q-value	Pathway ID
Glucosinolate metabolism	10 (1.28%)	25 (0.12%)	1.477425e-08	1.743362e-06	ko00966
Riboflavin metabolism	7 (0.89%)	34 (0.17%)	0.0002860938	1.687953e-02	ko00740
2-Oxocarboxylic acid metabolism	13 (1.66%)	118 (0.59%)	0.0007007765	1.687953e-02	ko01210
Phenylpropanoid biosynthesis	30 (3.83%)	424 (2.11%)	0.00130124	3.838658e-02	ko00940
Biosynthesis of secondary metabolites	106 (13.52%)	2078 (10.34%)	0.002334762	5.510038e-02	ko01110
Metabolic pathways	183 (23.34%)	3987 (19.83%)	0.007615617	1.011071e-01	ko01100
Circadian rhythm—plant	17 (2.17%)	238 (1.18%)	0.01232007	1.346494e-01	ko04712
Plant hormone signal transduction	39 (4.97%)	691 (3.44%)	0.01369316	1.346494e-01	ko04075
Protein processing in endoplasmic reticulum’	24 (3.06%)	462 (2.3%)	0.09507395	3.739575e-01	ko04141
MAPK signaling pathway-plant	24 (3.06%)	603 (3%)	0.4892315	9.284486e-01	ko04016
Starch and sucrose metabolism	22 (2.81%)	568 (2.83%)	0.5446477	9.284486e-01	ko00500

Its products are targeting soluble phenylpropanoid biosynthesis. WAK2 gene (AT1G21270, Ko4733) was significantly up-regulated by CAG. It is one of the wall-associated kinase (WAK) gene families in *Arabidopsis thaliana* that are receptors-like proteins associated to cell wall and required for cell expansion [22]. WAK2 gene is required to activate pectin of several genes in protoplasts which are involved in cell wall biogenesis [23]. The MIOX4 gene (AT4G26260, K00469) was slightly upregulated by CAG. It is one of the four MIOX genes in *Arabidopsis* (MIOX1, AT1G14520, MIOX2:AT2G19800, MIOX4 and MIOX5:AT5G56640) which converts MI to D-GlcUA and activates D-GlcUA into UDP-GlcUA in plant cells serving as a sugar precursor for plant cell walls [24]. MAPK signaling pathway was reported to regulate numerous cellular processes, including overcoming biotic stress [25]. AT4G36220 (K09755, ferulic acid 5-hydroxylase 1) was noticed to be up regulated under CAG treatment and was assigned to the three pathways ko00940, ko01100 and ko01110.

### Transcription factors (Tfs)

**Fig 7:**
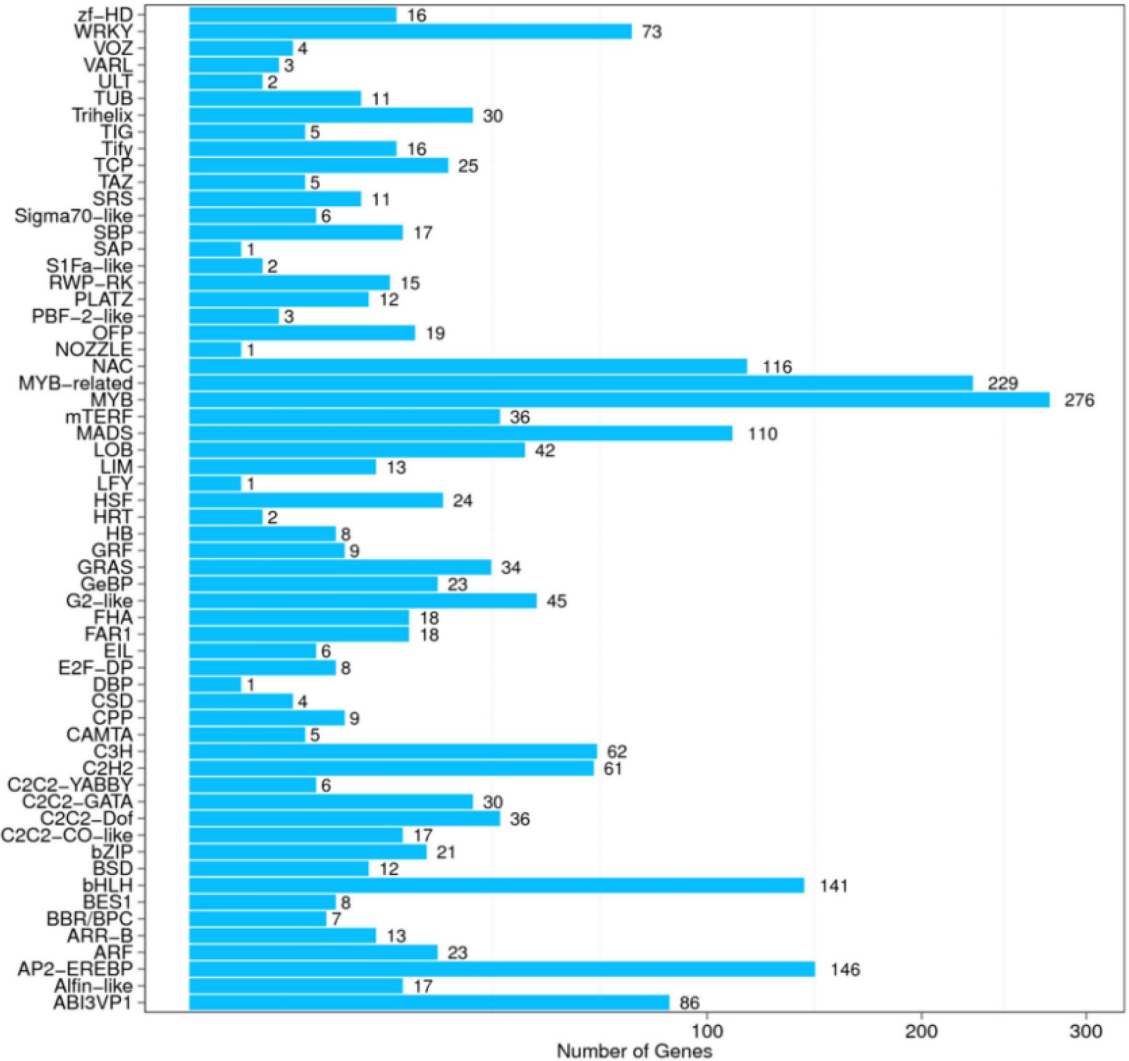
Gene classification on Tf Family.

A total of 2000 Tfs were identified in *Arabidopsis thaliana* calli during CAG treatment, among which 240 TFs are differentially expressed. These TFs were classified into 60 different common families (Fig 7). Among these families, the MYB family was the largest group (276, 13.8%), followed by MYB-related family (229, 11.45%), the AP2_EREBP family present (146, 7.3%) and the bHLH family 141, 7.05% from total identified TFs.

### Quantitative real-time PCR validate the RNAseq results

**Fig 8:**
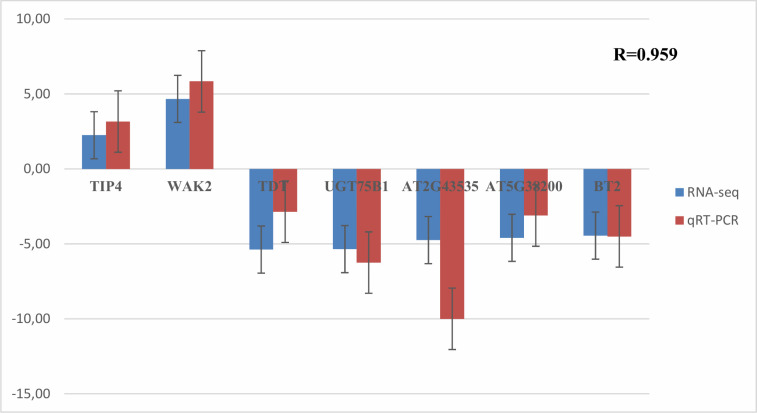
qRT-PCR validations of RNAseq results. Seven genes were randomly selected from the DEGs (blue columns) from the RNA-Seq data and were analyzed for differential expression changes (red columns) of the genes. The results were the average of three biological replicate samples in duplicate.

To check the RNA-Seq data, we randomly chose seven genes from the DEGs and used Primer3 software (version 0.4.0) to design specific primers for these genes (S1 Table). Three biological repetitions from each sample, Control and treated one, and two technical repetitions for each repetition have been run. Actine was used as a reference gene. Delta Delta ct method (ΔΔct) was used to calculate relative fold change values. Quantitative real-time PCR analyses exhibited that the relative expression patterns of the chosen genes were consistent with RNA-Seq data with a correlation coefficient of 0.95 between qRT-PCR and RNASeq (Fig 8). The results demonstrated that our RNA-Seq data are reliable.

## Discussion

Next-generation sequencing technologies are powerful tools to highlight the new genes and their involvement in biochemical pathways. No research work has studied CAG on plants generally or under specific conditions. Using RNA-seq, which is a beneficial way to reach the transcriptome data of plant tissue or cell subjected to particular conditions, our research provides a broad-spectrum study of the transcriptomic response to CAG in calli. In the GO classification, all genes, including DEGs, were revealed to be classified into three main categories; Molecular function (MF), Cellular Component (CC), and Biological Process (BP) containing 46 annotated functional subcategories. In the present work, CAG treatment resulted in no remarkable effect on callus growth index (preliminary results), although 1045 DEGs were obtained between the control and treated sample. DEGs data authenticity has been validated by qPCR analysis. DEGs of the most enriched GO terms for up-regulated and down-regulated were almost the same. The three categories are presented by up and down-regulated genes, with two exceptions; signal transducer activity (GO: 0039562) and nucleoid (GO: 0009295) subcategories, which are presented, respectively, to molecular function and cellular component categories of down-regulated genes. The DEGs are implicated in all subcategories, and the number of the involved down-regulated genes is more important than the up-regulated ones. Using KEGG analysis, most of DEGs were assigned to KEGG pathways. The analysis of their functions and the associated metabolic pathways revealed that DEGs were involved in macromolecules metabolisms pathways, transcription, replication and repair, and environmental adaptation and hormone signal transduction pathways. An important gene number has annotated some pathways, but few of them are differentially expressed; Ten DEGs were annotated to plant-pathogen interaction while in total 578 genes were annotated to this pathway, and only one gene is up-regulated. Six hundred ninety-one genes were assigned to the Plant-hormone signal transduction pathway, but among them, only 35 are found to be differentially expressed in the treated sample. The transcriptional response of the application of CAG on *Arabidopsis thaliana* calli has driven our research work into different signaling pathways. Based on KEGG analysis, the most significant pathway is glucosinolate (GSL) biosynthesis with ten differentially expressed genes which are AT3G19710 (BCAT4), AT5G23010 (MAM1), AT5G23020 (MAM3), AT1G16410 (CYP79F1), AT1G16400 (CYP79F2), AT4G13770 (CYP83A1), AT3G58990 (IPMI1), AT2G43100 (IPMI2), AT5G14200 (IMD1) and AT2G22330 (CYP79B3), that are involved mainly in the synthesis of glucosinolate (GSL) by deamination of Methionine to 2-oxomethylthiodecanoic acid and then to 8-methylthio-octyl-glucosinolte. It is known that plants have a reduced content of GSLs, very important in their defense against biotic stresses and in their nutritional values. Our study showed that CAG down-regulated all the previously cites genes. However, in other studies these genes had another type of regulation under different conditions. Actually, effect of different selenium and sulfur concentrations on this pathway has been studied. It was shown that BCAT4, MAM1, MAM3, and CYP79F1 in cabbage head in outer foliage leaf were up-regulated by exogenous Se and S treatment under concentrations of 50 μmol/L and 100 μmol/L, supporting the fact that S and Se metabolism interact with the biosynthesis of GSL including glucoraphanin, a major a major aliphatic GSL in cruciferous plants and the precursor of the anticancer compound sulforaphane. While the same concentration had no effect on other genes belonging to the same gene family like UGT74B1 and UGT74C1. In the same context, reported by the same authors, other studies have been shown that Se fertilizer does not affect or decrease the GLS contents [26]. This decrease could be caused by the negative effects of Se amino acids incorporation into proteins antagonistically affects GLS biosynthesis [27]. Hence the CAG effect on the GSL biosynthesis genes regulation might be caused by the 1μM CAG treatment concentration that it had an antagonist effect of the concerned genes and a lower concentration might affect differently. Plant-Hormone signaling transduction is one of the most enriched pathways after CAG treatment. Thirty-six differentially expressed genes have been registered, which means 4.97% from the total DEG number (Table 2). Specifically, the two genes AtbHLH38 (AT3G56970) and AtbHLH39 (AT5G04150) that belong to the basic helix-loop-helix (bHLH) gene family and their closets homologs, AtbHLH100 (AT2G41240) and AtbHLH101 (AT3G56980) which are termed subgroup Ib BHLH genes, regulated in leaves and roots in different nutritional variations as well as in mutant backgrounds and split-root situations associated to iron deficiency [28]. These genes are known as iron deficiency-regulated transcription factors and represent one of the enriched gene groups, AtbHLH100, AtbHLH101, AtbHLH38 and AtbHLH39 are up-regulated by 1.2, 2.8, 7.9, and 1.8 fold, respectively comparing to the control sample. These genes seem to be strongly up-regulated under Fe deprivation conditions in roots [29]. AtbHLH101 has also been shown to be up-regulated in response to the allelochemical L-Dopa, compound known to be an active allelochemical that decreases root growth of several plant species [30]. These facts might suggest the involvement of CAG in iron regulation and/or other mechanisms involved in root growth. Five genes belong to the heat shock proteins family and involved in the protein processing in the endoplasmic reticulum pathway.; HSP90.1 (AT5G52640), HSP17.6A (AT5G12030), HSP101 (AT1G74310), HSP17.4 (AT3G46230) and Hsp70b (AT1G16030), were up-regulated under CAG treatment. Heat shock proteins (HSPs) are known to be stimulated by high heat and prevent the aggregation of heat-sensitive proteins but play as chaperones a crucial role in conferring biotic and abiotic stress tolerance and enhance plant immunity by the accumulation of PR proteins under different stresses [31, 32]. HSP101 and HSP17.6A were among the up-regulated genes that have been identified after an excess of Ca^2+^ in *A*. *thaliana*, however, the increase in transcript abundance was not significant [33]. The fact that CAG increases HSPs expression means that Cycloastragenol might stimulate the stress tolerance process indirectly. Riboflavin metabolism has been significantly affected by CAG application. We registered seven differentially expressed genes, all down-regulated; AT2G01880 (PAP7), AT1G14700 (PAP3), AT4G29700, AT2G01890 (PAP8), AT4G29690, AT1G25230 (OPT3), AT2G38740. However, it has been proved that one of this genes family, OPT3, which is an oligopeptide transporter involved in metal homeostasis especially iron homeostasis is up-regulated by cadmium (Cd) suggesting a possible involvement in the management in iron transfer and/or distribution [34], however OPT3 is reported to encode Fe transporter functioning in stem phloem and is a major Cd tolerance gene in *A*. *thaliana* [35] which can lead to consider CAG to be potentially involved in Cd tolerance mechanisms. Interestingly, a subset of four unknown proteins was strongly up-regulated by CAG in calli; At1G47400 and AT1G47395, described both as hypothetical proteins, At2G30766; belongs to cytochrome P450 family and AT1G13609 which seems to be part of Defensin-like (DEFL) family protein. The last two genes have also been proved that they are up-regulated under Fe deficiency stress conditions but in leaves [36]. Fe is known as a component of the photosystems and essential for photosynthesis. Thus these two genes work diversely with photosynthesis which leads to suggest that CAG has an antagonist effect on this mechanism.

## Conclusion

In this work, we carried out the first transcriptomic analysis in *A*. *thaliana* under Cycloastragenol treatment through RNAseq. More than 40000 genes have been identified out of which around 22000 were differentially expressed. Multiple TFs were also identified and several protein kinases were regulated by CAG. The identified genes are part of significant physiological processes, signaling/metabolic pathways and regulatory networks. The differentially expressed genes and their corresponding pathways would be useful to expand our knowledge of CAG effects in *A*. *thaliana* specifically and in plants generally and then might be used to establish breeding programs related to biotic and/or abiotic stresses.

## Supporting information

S1 Fig Plant hormone signal transduction pathway in CAG-treated *A*. *thaliana* calli. (PDF)

S2 Fig MAPK signaling pathway-plant pathway in CAG-treated A. thaliana calli. (PDF)

S3 Fig Starch and sucrose metabolism pathway in CAG-treated A. thaliana calli. (PDF)

S4 Fig Protein processing in endoplasmic reticulum pathway in CAG-treated A. thaliana calli. (PDF)

S1 Table Primer sequences of the genes used in qRT-PCR analysis. (PDF)
