# Supplementary material for: Transcriptomic analysis reveals responses to Cycloastragenol in Arabidopsis thaliana
Source: PLoS One. 2020 Dec 10;15(12):e0242986. doi: 10.1371/journal.pone.0242986 (PMC7728452; doi:10.1371/journal.pone.0242986)
Supplement: S4 Fig — (PDF) [file pone.0242986.s004.pdf]

Transcriptomic Analysis reveals responses to Cycloastragenol in *Arabidopsis thaliana*

Wissem Mhiri[1¶*], Merve Ceylan[3&], Neslihan Turgut-Kara[2&], Barbaros Nalbantoğlu[1], Özgür Çakır[2¶*]

[1]Yıldız Technical University, Faculty of Art & Science, Chemistry Department, 34220, Istanbul, Turkey
[2]Istanbul University, Institute of Science, Program of Molecular Biology and Genetics, Istanbul, Turkey
[3]Istanbul University, Faculty of Science, Department of Molecular Biology and Genetics, 34134, Istanbul, Turkey

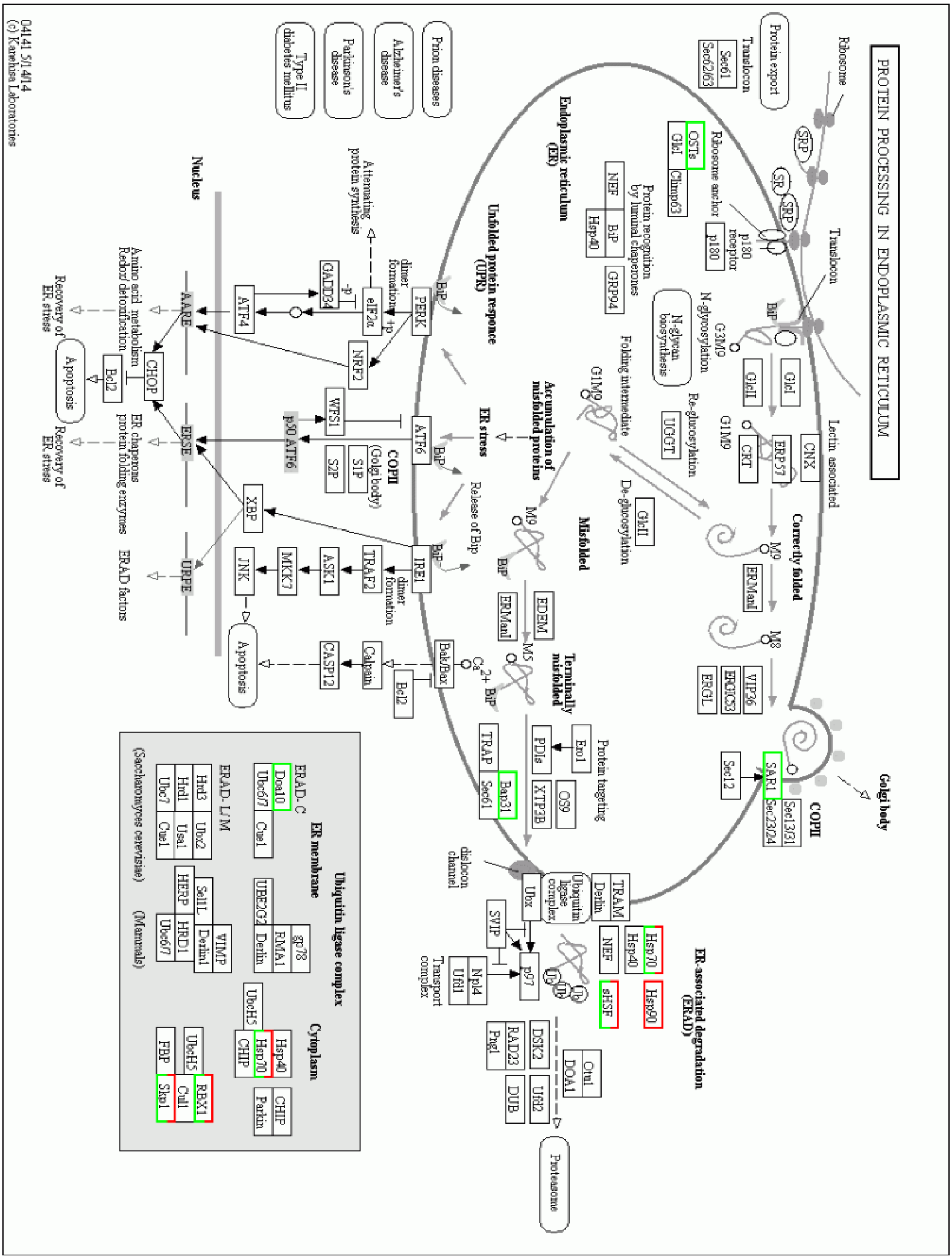

S4 Fig. Protein processing in endoplasmic reticulum pathway in CAG-treated *A. thaliana* calli

Red boxes and green boxes represent up-regulated and down-regulated genes, respectively.
