# Supplementary material for: Transcriptomic analysis reveals responses to Cycloastragenol in Arabidopsis thaliana
Source: PLoS One. 2020 Dec 10;15(12):e0242986. doi: 10.1371/journal.pone.0242986 (PMC7728452; doi:10.1371/journal.pone.0242986)
Supplement: S1 Table — (PDF) [file pone.0242986.s005.pdf]

S1 Table. Primer sequences of the genes used in qRT-PCR analysis

Gene Names	Sequences
**AT5G47560 (TDT)**	Forward 5'-CTTGTTATTGGGAGCTGGATTC-3'<br>Reverse 5'-GCGTTGTTTGAAGTGAACTCTG-3'
**AT1G05560 (UGT75B1)**	Forward 5'-TTCCCTCTGTTCATCTCTGGAT-3'<br>Reverse 5'-GTGTTGGAAGGTGAGAGGAAAG-3'
**AT2G43535**	Forward 5'-CTTCGCCATCTTTATCATCCTC-3'<br>Reverse 5'-AATATCTTAGGCGCACAGAAGC-3'
**AT5G38200**	Forward 5'-GCTTGTGGTGGGACTCTTTATC-3'<br>Reverse 5'- ACCAGGAATGAAGTGGACTGTT-3'
**AT3G48360 (BT2)**	Forward 5'-CATCTTTTAGCGTTGTCTCACG-3'<br>Reverse 5'- TTCAGTCTGCTCAACGGTCTTA-3'
**AT2G25810 (TIP4)**	Forward 5'-CTATTGTGGATCCGAAGAAAGG-3'<br>Reverse 5'- GTCCAGTTTCCAGAGACCAAAG-3'
**AT1G21270 (WAK2)**	Forward 5'-TTTCACAACCATCCTACTGTGC-3'<br>Reverse 5'- TGCAAGTCTTGTCTCCGATAGA-3'
**Actin**	Forward 5′-TGCTGACCGTATGAGCAAA-3′<br>Reverse 5′-CTCCGATCCAGACACTGTA-3′
